# Evaluation of Healthy Canine Conjunctival, Periocular Haired Skin, and Nasal Microbiota Compared to Conjunctival Culture

**DOI:** 10.3389/fvets.2020.00558

**Published:** 2020-08-27

**Authors:** Kayla C. Banks, Elizabeth A. Giuliano, Susheel B. Busi, Carol R. Reinero, Aaron C. Ericsson

**Affiliations:** ^1^College of Veterinary Medicine, University of Missouri, Columbia, MO, United States; ^2^Department of Veterinary Medicine and Surgery, College of Veterinary Medicine, University of Missouri, Columbia, MO, United States; ^3^Luxembourg Centre for Systems Biomedicine, University of Luxembourg, Esch-sur-Alzette, Luxembourg; ^4^Comparative Internal Medicine Laboratory, University of Missouri, Columbia, MO, United States; ^5^Department of Veterinary Pathobiology, University of Missouri Metagenomics Center, University of Missouri, Columbia, MO, United States; ^6^Department of Veterinary Pathobiology, College of Veterinary Medicine, University of Missouri, Columbia, MO, United States

**Keywords:** ocular surface, periocular haired skin, nasal, microbiota, operational taxonomic unit (OTU), 16S rRNA sequencing, diversity, culture

## Abstract

Next-generation sequencing (NGS) methods have been used to identify a diverse ocular surface (OS) microbiota in humans. These results have highlighted limitations in microbial detection via traditional culture-based techniques. The OS has mechanisms such as tear film and mechanical blinking, which may aid in preventing adherence and colonization of microbes, suggesting that only low populations of microbes may reside on the OS. Additionally, closely related tissues to the OS are exposed to a similar array of microbes, but demonstrate different defense mechanisms. Information regarding concordance of microbial communities of the OS and nearby tissues is lacking. Our study purposes were to (1) characterize the conjunctival microbiota of healthy dogs, (2) compare the conjunctival microbiota to the periocular haired skin and distal nose, and (3) compare the bacteria identified by culture to NGS of the healthy canine conjunctiva. Here, NGS was used to evaluate samples from 25 healthy adult dogs of the conjunctiva, periocular haired skin, and distal nose. Additional samples were collected from each dog for traditional conjunctival culture. The 16S rRNA gene amplicon libraries were evaluated for coverage, relative abundance, richness, and diversity. Site-dependent similarities evaluated using principal coordinate analysis (PCoA) and PERMANOVA demonstrated relatedness in community compositions between sites. The conjunctiva of healthy dogs yielded a rich and diverse microbiota based on NGS. While some regional continuity was noted, microbial communities of the conjunctiva, periocular haired skin, and nose were significantly different from each other. Comparatively, traditional culture markedly underestimated the number of bacterial taxa present on the healthy canine OS. Findings suggest similarities in nasal and conjunctival microbial communities, which may be a result of similarities in mucosal immunity and anatomic connection via the nasolacrimal system. Further investigation using NGS into changes of the composition of bacterial communities in disease is warranted.

## Introduction

The ocular surface has a wide array of mechanisms which prevent adherence and ensuing infection of microorganisms. These functional and immunologic processes range from antimicrobial peptides [e.g., secretory immunoglobulin A (IgA), components of complement, lipocalin, lysozyme, lactoferrin, etc.] within the tear film to mechanical blinking to tight junctions between the epithelial cells of the cornea (1, 2). As such, it is not surprising that OS culture sometimes yields negative results for bacterial growth in both healthy and disease states. In the early 2000s, alternatives to traditional culture were developed using molecular techniques to profile microbial communities based on conserved regions in bacterial genomes. These culture-independent techniques (NGS), which analyze microbial DNA extracted from a sample collected from the desired area of study (e.g., feces, conjunctiva, skin, blood), allow for characterization of complex microbial communities using genomic databases for identification. Aberrant alterations in the microbiota, termed dysbiosis, are implicated in both cause and effect of systemic disease, with the human gut being a large focus in biomedical research [e.g., *Clostridium difficile*-associated diarrhea, colorectal cancer, obesity; (3–5)].

Since the initial undertaking of the Human Microbiome Project (6, 7) which reported the presence of rich and diverse microbiota among a variety of anatomic sites, research has expanded into parts of the human body previously thought to be devoid of microbial presence (8, 9). As the use of culture-independent techniques expands, investigations of the OS have concluded that there are diverse microbial populations present on the healthy human eye (10–13). The influence of these microbial communities on OS health remains unclear, but changes in community structure have been documented with OS and adnexal disease states (14–17).

In homeostatic states, many organisms likely serve as commensal microbes playing a role in maintaining health; however, perturbation of the healthy ocular or extraocular microbiota may cause or contribute to disease pathogenesis (18). Minimal research has been performed on common veterinary species to identify the presence and diversity of microbial populations and whether correlations can be made in order to establish an animal model for human disease. With that said, similarities in diagnosis and treatment exist for many OS diseases (e.g., infectious and inflammatory keratitis, keratoconjunctivitis sicca, blepharitis) between veterinary species and humans. Further investigation of veterinary species microbiota may reveal an excellent animal model for evaluating the effects of novel therapeutic strategies on the OS microbiota. In order to further understand the effects of these contributing factors, here NGS of the OS are being paired with periocular haired skin and the distal nasolacrimal puncta. A direct comparison of microbial community compositions of the OS and closely associated anatomic structures may provide insight to how the functional and immunologic mechanisms impact the presence and stability of microbial communities on the ocular surface.

We hypothesized that NGS would identify a rich and diverse microbiota of the healthy canine conjunctiva and demonstrate some commonalities with the nasal and periocular microbiota. Additionally, we expected NGS to yield a richer and more diverse microbial community compared to traditional conjunctival culture. To characterize the OS bacterial microbiota in normal dogs, samples were collected from the inferior-nasal fornix of the conjunctiva from 25 healthy dogs. To investigate potential correlations of microbial communities between nearby anatomic sites, additional samples were collected from the nose at the level of the distal nasolacrimal puncta and the ventral periocular haired skin. All of these samples went through standard DNA extraction methods for low-biomass samples followed by 16S rRNA amplicon sequencing (19). Lastly, to provide a direct correlation to traditional methods using matched samples, a second set of samples was collected from the same location within the conjunctiva for aerobic culture.

## Methods

### Ethics Statement

The study was approved by the University of Missouri Institutional Animal Care and Use Committee (Animal Use Protocol #9232) and was conducted in accordance with the ARVO Statement for the Use of Animals in Ophthalmic and Vision Research.

### Dogs

All dogs were recruited from the general population presenting for basic veterinary care or belonging to students and staff of the University of Missouri Veterinary Health Center. Twenty-five clients that owned medium-sized dogs between 15 and 25 kg and 1–8 years of ages were selected (demographic data provided in Supplementary Table 1). Dogs were free of systemic and ophthalmic disease. Dogs were excluded if they were fractious or brachycephalic or received any antimicrobials or ophthalmic medications within 2 months of sample collection. Evaluation and sample collection were performed in April 2019 in all dogs. All dogs had a complete evaluation of the anterior segment of the eye by slit-lamp biomicroscopy (SL-17, Kowa Optimed Inc., Torrance, CA), and the posterior segment of the eye by indirect ophthalmoscopy (Vantage Plus Wireless Headset, Keeler Instruments Inc., Malvern, PA) prior to sample collection. Additionally, a routine minimal ophthalmic database was performed a minimum of 2 h following sample collection to prevent contamination or dilution of the sample. This included Schirmer tear test measurements (Schirmer Tear Test, Merck Animal Health, Summit, NJ 07901), fluorescein staining (Ful-Glo 0.6 mg Fluorescein Sodium Ophthalmic Strips, Akorn Inc., Lake Forest, IL 60045), and tonometry (TONOVET tonometer, Jorgensen Laboratories Inc., Loveland, CO).

### Sample Collection

One drop of 0.5% proparacaine (proparacaine hydrochloride 0.5% ophthalmic solution, Alcon Laboratories, Inc. Fort Worth, Texas 76134) was placed on the OS to provide topical anesthesia. Samples were collected with sterile culturette tips in a repetitive sweeping motion 10 times from the following sites: inferior conjunctival fornix of the left eye, left nares at the level of the distal nasolacrimal puncta, and periocular haired skin approximately 1 cm distal to the inferior eyelid margin. Swab samples were collected into sterile 1.5-mL Eppendorf tubes by cutting the distal tip of the swab off into a tube containing lysis buffer as previously described (500 mM NaCl, 50 mM Tris–HCl, 50 mM EDTA, 4% SDS) and stored immediately at −80°C until the time of DNA extraction and amplification (20). An additional sample was collected from the inferior nasal conjunctival fornix then immediately submitted to the University of Missouri Veterinary Medical Diagnostic Laboratory for aerobic culture.

### Bacterial Culture

Culture methods were performed using established techniques of the University of Missouri Veterinary Medical Diagnostic Laboratory. Swabs were plated directly onto blood and MacConkey agar for aerobic growth (at 35°C in ambient air) then placed into thioglycolate broth at 35°C. Media were examined at 24 and 48 h; if no growth was evident at 48 h, plates and broth were discarded and culture was reported negative. If positive growth was noted, bacterial identification was performed using MALDI-TOF (Matrix Assisted Laser Desorption/Ionization-Time of Flight: Bruker Daltonics, Inc., 40 Manning Road, Manning Park, Billerica, MA 01821).

### DNA Extraction

Prior to processing, swabs were thawed and removed from lysis buffer. Lysis buffer was transferred to bead tubes from PowerFecal kits (QIAGEN, Crawley, UK), and DNA extraction proceeded according to the manufacturer's instructions, with the exception that, rather than performing the initial homogenization of samples using the vortex adapter described in the protocol, samples were homogenized in the provided bead tubes using a TissueLyser II (Qiagen, Venlo, Netherlands) for 3 min at 30/sec, before proceeding according to the protocol and eluting in 100 μL of elution buffer (QIAGEN, Crawley, UK). DNA yields were quantified via fluorometry (Qubit 2.0, Invitrogen, Carlsbad, CA) using quant-iT BR dsDNA reagent kits (Invitrogen, Carlsbad, CA). Two additional unused swabs were exposed to air, placed in sterile extraction buffer, and then processed and sequenced alongside experimental samples as negative controls.

### 16S rRNA Library Preparation and Sequencing

Extracted DNA was processed at the University of Missouri DNA Core Facility. Bacterial 16S rRNA amplicons were constructed via amplification of the V4 region of the 16S rRNA gene with universal primers (U515F/806R) previously developed against the V4 region, flanked by Illumina standard adapter sequences (21, 22). Oligonucleotide sequences are available at proBase (23). Dual-indexed forward and reverse primers were used in all reactions. PCR was performed in 50-μL reactions containing 100 ng metagenomic DNA, primers (0.2 μM each), dNTPs (200 μM each), and Phusion high-fidelity DNA polymerase (1 U). Amplification parameters were 98°C^(3:00)^ + [98°C^(0:15)^ + 50°C^(0:30)^ + 72°C^(0:30)^] × 25 cycles + 72°C^(7:00)^. Amplicon pools (5 μL/reaction) were combined, thoroughly mixed, and then purified by addition of Axygen Axyprep MagPCR clean-up beads to an equal volume of 50 μL of amplicons and incubated for 15 min at room temperature. Products were then washed multiple times with 80% ethanol and the dried pellet then resuspended in 32.5 μL EB buffer, incubated for 2 min at room temperature, and then placed on the magnetic stand for 5 min. The final amplicon pool was evaluated using the Advanced Analytical Fragment Analyzer automated electrophoresis system, quantified using quant-iT HS dsDNA reagent kits, and diluted according to Illumina's standard protocol for sequencing on the MiSeq instrument.

### Informatics

Primers were designed to match the 5′ ends of the forward and reverse reads. Cutadapt (version 2.6; https://github.com/marcelm/cutadapt) was used to remove the primer from the 5′ end of the forward read. If found, the reverse complement of the primer to the reverse read was then removed from the forward read as were all bases downstream. Thus, a forward read could be trimmed at both ends if the insert was shorter than the amplicon length. The same approach was used on the reverse read, but with the primers in the opposite roles. Read pairs were rejected if one read or the other did not match a 5′ primer, and an error rate of 0.1 was allowed. Two passes were made over each read to ensure removal of the second primer. A minimal overlap of 3 with the 3′ end of the primer sequence was required for removal.

The Qiime2 dada2 plugin (version 1.10.0) was used to denoise, de-replicate, and count ASVs (amplicon sequence variants), incorporating the following parameters: (1) forward and reverse reads were truncated to 150 bases, (2) forward and reverse reads with number of expected errors higher than 2.0 were discarded, and (3) Chimeras were detected using the “consensus” method and removed. R version 3.5.1 and Biom version 2.1.7 were used in Qiime2. Taxonomies were assigned to final sequences using the Silva.v132 database, using the classify-sklearn procedure.

### Statistics

Data associated with richness and α-diversity were first tested for normality using the Shapiro–Wilk method. In both cases (Chao1 and Shannon indices), normality failed, and group differences were tested using a non-parametric Kruskal–Wallis analysis of variance (ANOVA) on ranks. *Post-hoc* pairwise comparisons were performed using Tukey's test. The above analyses were performed using SigmaPlot 14.0 (Systat Software Inc., Carlsbad, CA).

Similarities within and between sample sites (i.e., β-diversity) were visualized using principal coordinate analysis (PCoA), using the relatively unweighted Jaccard and weighted Bray–Curtis indices. Site-dependent differences in β-diversity were tested using multivariate permutational ANOVA (PERMANOVA), again using both Jaccard and Bray–Curtis similarities as appropriate. For all PERMANOVA testing, *p*-values are provided alongside *F*-values. These multivariate analyses were performed using open-access Past 3.26 software download August 2019 (Reference: Hammer O. Past 3.x 2019, available from: http://folk.uio.no/ohammer/past/).

## Results

### Conjunctival Microbiota Is Comparable in Richness and Diversity to That of Distal Nasal Lacrimal Puncta and Periocular Haired Skin

**Figure 1:**
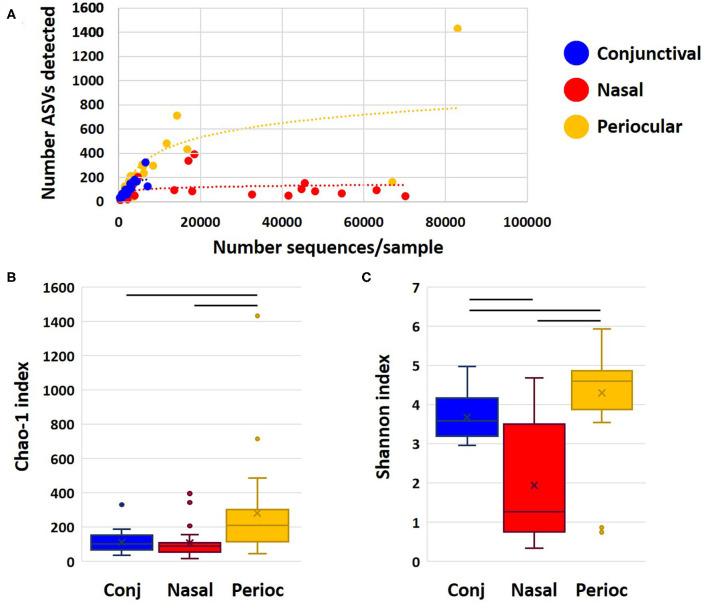
Dot plot showing rarefaction of detected richness and sample coverage for each sample site **(A)**, and Chao1 **(B)**, and Shannon **(C)** indices as estimates of true richness and α-diversity, respectively. Bars indicate *p* < 0.05, Kruskal–Wallis ANOVA on ranks.

Samples returned a variable number of high-quality sequences, with the highest mean (±SEM) count obtained from nasal samples (11,825 ± 3,709), and, as expected, lower counts from periocular haired skin (3,903 ± 3,917) and conjunctival (2,412 ± 351) swabs. Control swabs returned 28 and 42 sequences. Two other samples (one conjunctival and one periocular) yielded similarly low read counts, and all samples from those two dogs were removed from subsequent analyses. Despite the greater community coverage of nasal samples, comparison of coverage to the number of distinct amplicon sequence variants (ASVs) detected in each group demonstrated a plateau in nasal samples suggesting that those communities were well-represented in the current dataset (Figure 1A). In contrast, rarefaction of periocular and conjunctival samples suggested that increased coverage might reveal additional rare ASVs. The Chao1 index is an estimate of predicted true richness based on the detected richness and the number of ASV singlets and doublets in the dataset. Despite the greater coverage of nasal samples, periocular samples were found to harbor significantly richer communities than samples from the other sites (Figure 1B). Similarly, comparison of the Shannon index, an indicator of α-diversity that is extremely robust to poor coverage, revealed significantly greater diversity among the periocular samples than the other two sample sites, and greater diversity in conjunctival samples compared to nasal (Figure 1C). Eliminating the unlikely possibility that these differences were due to differential coverage, repeat analysis with sequence data subsampled to a uniform read count of 1,461 sequences per sample revealed the same differences (Supplementary Figure 1). Thus, these data generally support the concept of a rich conjunctival microbiota, comparable to that of nasal and periocular sites.

### Microbial Community Structure of Conjunctiva, Periocular Haired Skin, and Distal Nasal Lacrimal Puncta Is Similar Within Sites but Differs Between Sites

**Figure 2:**
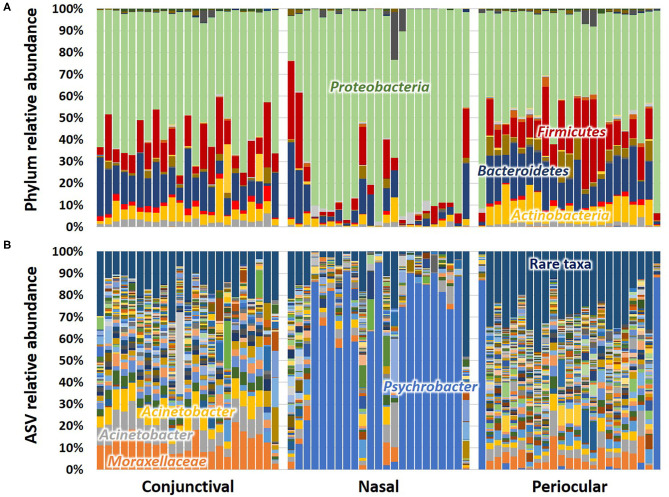
Stacked bar charts showing the taxonomic composition of all 23 samples at each site, annotated to the level of phylum **(A)** and amplicon sequence variant (ASV) **(B)**. Dominant taxa are labeled directly on the chart.

Subjective assessment of the community structure at each site at the level of the phylum (Figure 2A) reveals similarities in the detected taxonomies, albeit with clear site-dependent differences in their relative abundance. For example, while *Actinobacteria, Bacteroidetes, Firmicutes*, and *Proteobacteria* represent the four dominant phyla at all sample sites, the extent to which *Proteobacteria* overshadows the other three phyla varies between sites. Annotated to the level of ASV, there is a similar general agreement, particularly between conjunctival and periocular samples, with regard to community composition (Figure 2B). Again however, there are several subtle differences in the relative abundance of select ASVs. Most notably, most of the *Proteobacteria* DNA seen in the nasal samples annotated to the genus *Psychrobacter*, fast-growing aerobes recognized to colonize mucosal surfaces. Other dominant genera included *Acinetobacter* and undetermined *Moraxellaceae*, both repeatedly detected at 5–10% or higher, particularly in conjunctival and periocular samples, and ubiquitous in all samples.

### Conjunctiva Is More Similar to Distal Nasal Lacrimal Puncta Than Adjacent Periocular Haired Skin in Terms of Microbial Community Membership

Much of the compositional differences are impossible to show in a stacked bar chart format, as evidenced by the substantial area comprising rare taxa from each sample in Figure 2B. To better characterize similarities in β-diversity, principal coordinate analysis (PCoA) and permutational multivariate ANOVA (PERMANOVA) were performed using multiple similarities (weighted Bray–Curtis and unweighted Jaccard). Unweighted similarities determine the “likeness” between two samples based on the number of shared ASVs between the two samples, in relation to the number of unique ASVs in either sample. Weighted similarities also ascribe “likeness” based on shared presence of taxa but also on similarity in the relative abundance of taxa (particularly dominant taxa).

**Figure 3:**
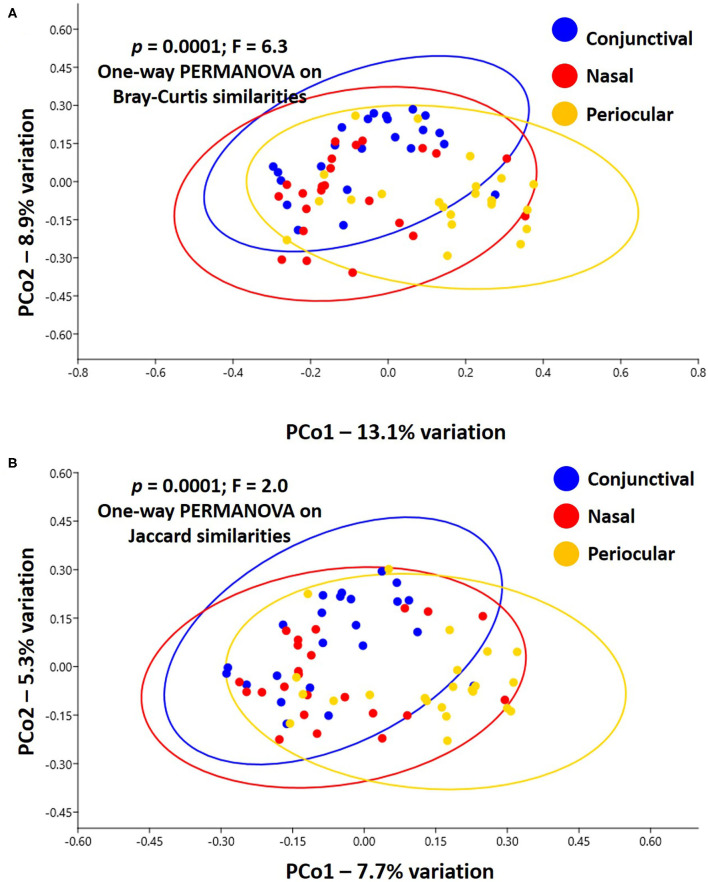
Principal coordinate analysis showing the β-diversity within and between sample sites, as determined using Bray–Curtis **(A)** or Jaccard **(B)** similarities. Ovals represent 95% confidence intervals. Results of PERMANOVA are given on each plot; legend at right.

Notably, PERMANOVA detected significant and substantial differences between all three sample sites based on both Bray–Curtis similarities (*p* = 0.0001; *F* = 6.3) and Jaccard similarities (*p* = 0.0001; *F* = 2.0). Pairwise comparisons and PCoA indicated that, based on the total dataset, conjunctival communities were distinct from both nasal and periocular communities regardless of the similarity metric used (Figure 3, Table 1). The same comparisons made using the data subsampled to a uniform coverage only strengthened these differences as reflected in Supplementary Figure 2.

**Table 1:** Results of permutational multivariate ANOVA (PERMANOVA) testing for differences in β-diversity among different sample sites, based on all sequences or a subsampled dataset, and using either Bray–Curtis or Jaccard similarities.

		**All sequences**		**Subsampled (1,461 reads)**	
		**Bray–Curtis**	**Jaccard**	**Bray–Curtis**	**Jaccard**
Overall	*p*	0.0001	0.0001	0.0001	0.0001
	*F*	6.3	2.0	14.0	3.3
Conjunctival vs. nasal	*p*	0.0001	0.0007	0.0001	0.0001
	*F*	9.9	1.7	23.5	3.4
Conjunctival vs. periocular	*p*	0.0002	0.0001	0.0001	0.0001
	*F*	3.5	2.4	6.9	3.0
Nasal vs. periocular	*p*	0.0001	0.0002	0.0001	0.0001
	*F*	5.4	2.0	13.1	3.5

*Main effects between all sample sites (overall) are followed by results of pairwise comparisons*.

**Figure 4:**
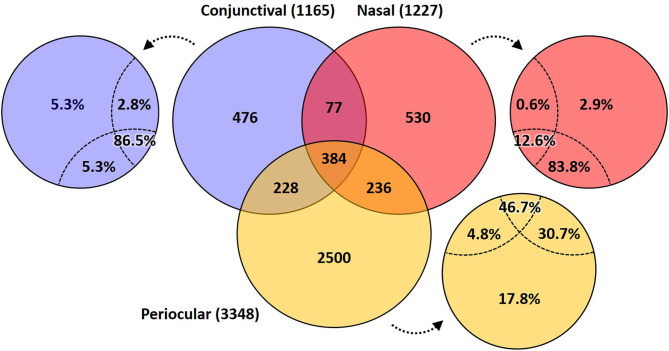
Venn diagram showing the number of ASVs detected in at least one sample from each site (i.e., conjunctival, nasal, and periocular), or a combination thereof. Small circles adjacent to the Venn diagram indicate the proportion of total DNA from that sample site represented by taxa in the indicated space.

**Figure 5:**
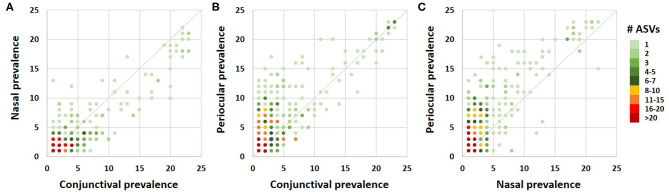
Correlation dot plots based on the 384 amplicon sequence variants detected in at least one sample from all three sample sites, comparing the prevalence in conjunctival and nasal samples **(A)**, conjunctival and periocular samples **(B)**, and nasal and periocular samples **(C)**. The color of each dot indicates the number of ASVs represented by that dot, legend at right.

A Venn diagram assessment of the 1,165, 1,227, and 3,348 ASVs identified in at least one of the conjunctival, nasal, or periocular samples, respectively supports the notion that there is greater than would be expected agreement between the conjunctival and nasal communities, despite the closer proximity of the periocular sample site. Specifically, of the 3,348 ASVs detected in at least one of the periocular samples, 612 (18.3%) were detected in at least one of the conjunctival samples. In contrast, of the 1,227 ASVs detected in at least one of the nasal samples, 461 (37.6%) were detected in at least one of the conjunctival samples (Figure 4). Upon closer inspection, however, it became apparent that the 476 ASVs that were unique to conjunctival samples, as well as the 77 ASVs found in conjunctival and nasal samples, and 228 ASVs found in conjunctival and periocular samples, represented relatively rare taxa found at extremely low relative abundance, and in only a handful of samples. In contrast, the 384 ASVs that were detected in at least one sample from all three sites represented 86.5, 12.6, and 46.7% of the microbial DNA detected in conjunctival, nasal, and periocular samples, respectively. The difference in those numbers is due to the fact that two ASVs annotated to the genera Psychrobacter and Pasteurella represented a combined 76.3 and 24.4% of sequences from nasal and periocular samples, respectively, but were not detected in a single conjunctival sample. Supplementary Table 2 shows the taxonomies of all ASVs identified in greater than half of the samples from any given site, 23 of them being detected at high prevalence in all three sites. Comparison of the prevalence of those 384 ASVs in each pairwise combination of sample sites again suggested a closer correlation between conjunctival and nasal samples in the prevalence of ASVs found in all three sample sites, relative to the other two pairings (i.e., conjunctival and periocular, and nasal and periocular; Figure 5). Moreover, there was considerable agreement between the different sample sites in those taxa detected in >50% of samples from each site (Supplementary Table 2). Thus, while there is likely transient colonization of the conjunctival surface from multiple sources, the present data demonstrate a substantial overlap of the canine conjunctival microbiota with that of nearby anatomical sites, particularly the distal nasal lacrimal puncta.

### Traditional Conjunctival Culture Underestimates Bacterial Taxa Present in Healthy Conjunctiva

Lastly, matched samples to those analyzed using 16S rRNA sequencing were subjected to a clinically based culture protocol to provide an estimate regarding the proportion of the conjunctival microbiota that is cultivable using standard practices. Aerobic culture of the inferior nasal conjunctival fornix was positive in 11/25 (44.0%) dogs. Of the positive cultures, 10/11 (90.9%) dogs had growth from enrichment broth only and one had light growth on direct culture. A single bacterial species was isolated on culture from 9/11 (81.8%) and two different bacterial species were isolated on culture for the remaining two dogs. Bacterial species isolated are shown in Supplementary Table 3. Overall, 13 organisms were isolated and 12 of these (92.3%) were Gram positive. The most commonly cultured genus was *Staphylococcus* spp. (6/13 isolates, 46.2%) followed by *Bacillus* spp. (5/13, 38.4%). A single organism was isolated from both *Paenibacillus* spp. and *Enterococcus* spp. genera.

While aerobic culture revealed between zero to two bacterial species, 16S rRNA sequencing identified between 6 and 850 (median, 237) unique taxa from the paired conjunctival samples. Subjects with a positive traditional culture (11/25) were identified, and within those 11 samples, 16S rRNA sequencing identified a range of taxa from 94 to 354 (median, 239). By comparison, subjects with a negative conjunctival culture were evaluated with 16S rRNA sequencing and had a range of taxa from 6 to 850 (median, 209).

## Discussion

Sequencing technology advancements have allowed for improved understanding of the vast diversity and importance of microbial communities in human and veterinary medicine. Utilization of these techniques for investigating the canine OS microbiota has been minimal (24, 25). Our study documents that the canine OS harbors a more rich and diverse microbial community than was previously recognized utilizing traditional culture-based techniques. Assessment of richness and alpha diversity by sample location suggests greater similarity of the conjunctival and nasal microbial communities, but lower richness and alpha diversity compared to periocular samples. The periocular, and to a lesser degree conjunctival samples, harbors large quantities of rare taxa. Evaluation of β-diversity reveals that all three sample locations have distinct microbial community composition; however, some degree of regional continuity is appreciated. β-Diversity measurements further support greater likeness of conjunctival and nasal communities.

Despite yielding a populous microbial community on the canine conjunctiva, coverage, and richness were lowest of all sampled locations. It is plausible that though the ocular surface has constant exposure to airborne debris and microbes, the immunologic properties and antimicrobial peptides of the tear film along with the mechanical motion of the eyelids aid in frequent clearance and turnover of microbial organisms from the OS (2). However, this concept has not been extensively evaluated in any species.

Significantly higher richness and α-diversity was observed on the periocular haired skin compared to both mucosal surfaces, which is consistent with previous findings (24). We postulate that difference in the local immune systems may play a role in these differences. The cutaneous immune system differs significantly from the mucosal surfaces as the epidermis serves primarily as a physical barrier with a more diffuse secondary immune defense [e.g., antimicrobial peptides and cytokines, Langerhans cells, and dermal dendritic cells; (26)]. Unlike the conjunctiva and nasal mucosa, the epithelium does not produce a protective mucin and have ciliated epithelial cells or numerous lymphoid aggregates in the lamina propria. The increased microbial richness in the periocular samples, especially of rare taxa, may represent transient environment bacteria and reside within this exposed area without stimulation of the local cutaneous immune system deep to the epithelial barrier. Additionally, as samples were collected in an area of skin covered by variable quantities of fur, the location may serve as a reserve for bacteria between the hair shafts and epithelium.

As we recognize the prevalence of rare taxa, it raises the question of the role of bacteria as “transient” vs. “colonizer.” We recognize that all microbes have the ability influence the inflammatory tone of a tissue via microbe-associated molecular patterns (27, 28). Many localized inflammatory conditions are believed to be due, in part, to increased exposure and alterations in the local immune system (e.g., exposure keratitis, allergic conjunctivitis, keratoconjunctivitis sicca). As our understanding of the microbial communities within this area is limited, it is challenging to discern how the interactions of transient vs. colonizing bacteria may alter the local immunity in health or disease conditions such as those listed above. Therefore, further evaluation of microbial communities with OS disease may provide suggestions of these potential associations. Further, it may suggest therapeutic strategies to target dysbiosis locally.

Proteobacteria was the most abundant phylum identified in all sampled locations. Major phyla detected in the nasal samples (*Proteobacteria, Firmicutes*, and *Bacteroidetes)* are consistent with previous studies (24, 29, 30). Conjunctival samples and periocular evaluations have not been extensively evaluated; however, parallels can be drawn for the community structures at the phylum though relative abundance is variable (24, 25). Prominent taxa detected in the conjunctiva (and to variable amounts in the periocular samples) when employing NGS included *Acinetobacter* spp. and *other Moraxellaceae* spp. Unsurprisingly, a frequent commensal organism of mucosal surfaces *Psychrobacter* spp. dominated the nasal microbiota of our subjects, which agrees with previous studies (19, 24, 30). The previously mentioned genera fall within the *Moraxellaceae* family, which are considered common commensal organisms of mammals. It should be noted that several specific species within this family have been identified as bacterial opportunists and of variable pathogenicity. *Acinetobacter* spp. are well-known bacteria, primarily found in soil but are implicated in some human opportunistic infections (31–33). *Acinetobacter* was identified as a nearly ubiquitous genus in two preliminary reports of the human conjunctival microbiota (34, 35). No speculation in physician literature has been made about why these genera are commonly found in the conjunctiva. Perhaps they represent environmental bacteria to which the ocular surface and periocular tissue are commonly exposed.

The relationship between nasal and conjunctival communities may be influenced by a number of factors including direct communication via the nasolacrimal drainage system, similarities in mucosal immunity, or as a function of host-mediated selection at these two mucosal surfaces. The ocular surface is covered by a thin tear film which is constantly produced. In health, the excess tear volume is flushed out via nasolacrimal drainage. Tears enter the superior and inferior canaliculi via puncta within the palpebral conjunctiva in the medial canthal region and then progress via gravity and capillary action to the lacrimal sac. The tears then continue along the narrow lumen of the nasolacrimal duct to the nasal puncta which opens approximately 1 cm inside the external nares at the ventral lateral nasal meatus. Tears may impact the local microbiota of both the nasolacrimal duct and the distal nose.

Mucosal surfaces contain physical and chemical barriers including a thin epithelium with tight junctions. The conjunctival and nasal mucosa secrete variable antimicrobial compounds (e.g., defensins, cathelicidins, IgA), produce mucous, and contain aggregates of lymphoid follicles within the lamina propria [e.g., MALT—mucosa-associated lymphoid tissue; (2, 26, 36)]. Within this mucosal surface, specialized epithelial cells (M cells) allow antigens to cross through the basal surface to the subcutaneous space where large quantities of antigen-presenting cells reside to process antigens. These cells then determine whether to deliver it to the local MALT, which is programmed for tolerance of non-pathogenic antigens. Further regional specialized lymphoid tissues have been recognized in the dog conjunctiva (CALT, conjunctiva-associated lymphoid tissue) (37) and within the lacrimal duct in humans (LDALT, lacrimal drainage-associated lymphoid tissue) (38), which may further modify the microbial communities. Therefore, while mucosal microbial communities of the conjunctiva and distal nose demonstrate likeness, minor differences between the mucosal microbial communities would be expected based on differences in tolerance in localized lymphoid tissue.

Conventional culture was performed here to subjectively compare the results of culture to NGS. Culture is utilized by the veterinary ophthalmology community to evaluate pathogenesis of progressive corneal disease (e.g., malacic ulcerative keratitis) and as a guide for therapy. Previous work using traditional culture has also been used for evaluation of the healthy canine ocular surface (1, 39–43). Here, we demonstrate that conventional methods markedly underestimated the bacterial taxa present in the healthy conjunctiva (0–2 cultivable species vs. up to 850 taxa using NGS). The most commonly cultured species of bacteria were *Bacillus* spp. and *Staphylococcus* spp. Despite the frequency of detection by conventional culture, the latter accounted for 1.6% of all reads in our conjunctival samples using NGS while the former was not detected at all, which provides similar results to a previous investigation of the conjunctival microbiota of a less variable subject population (25). Conversely, several taxa detected via NGS at high relative abundance were not detected using the described culture methods as would be expected. Primary examples include *Acinetobacter* and other members of the *Moraxellaceae* family, and Gram-negative aerobes reported to grow well on many standard media including MacConkey agar. Thus, NGS provides a more complete representation of the microbial communities in the healthy ocular surface as the majority of bacterial species are not cultivable (44–46). These data also serve as a cautionary tale regarding the reliance on culture when making clinical diagnosis and treatment decisions. Clearly, certain taxa are readily cultured from healthy eyes (representing false positives), while myriad other bacterial taxa, including Gram-negative aerobes ostensibly capable of causing inflammation were tissues are damaged, were detected via NGS but not grown on culture (representing false negatives). NGS is likely too expensive at present for routine use in the clinics, and results indicate only relative abundance of detected taxa, rather than absolute numbers. Thus, it is anticipated that taxa found to serve as diagnostic or prognostic indicators of disease in future studies would be translated into quantitative-PCR assays for use in the clinics.

Limitations to this study include the inability to discriminate between viable bacteria and residual DNA as well as to determine transient vs. colonizing bacteria at the sampled locations. Another inherent constraint of NGS is difficulty in determining absolute quantities of microbial populations, so direct comparison to culture-based colony-forming units is not applicable. Also, without culture, antimicrobial sensitivity cannot be performed. Therefore, this methodology cannot be readily substituted for traditional culture and susceptibility. Additionally, at this time NGS is not clinically applicable due to expense and time associated with processing and analyzing samples. Finally, there is no “normative” data that can be used as a reference to compare individual patients' results as there is intrinsic variation between individuals. Related to this, the current study is not adequately powered to perform regression analyses incorporating variable such as sex, haircoat length and type, breed, and other factors that may affect the relationship between microbial communities at each sample site.

Despite some limitations of NGS, the current study provides consequential information regarding healthy, client-owned dog's microbial communities of the OS and nearby anatomic sites. It suggests general trends in microbial community compositions of the sampled locations. These data provide the groundwork for future investigations on the role of the microbiota in ocular diseases. This study also provides a foundation of the healthy OS microbial community structure for future studies on effects of medical therapies such as anti-inflammatories, antibiotics, and immunomodulatory and immunosuppressive drugs.

In conclusion, canine conjunctiva yields a richer and more diverse population than was previously recognized. While there was some degree of regional continuity, microbial communities of the conjunctiva, periocular haired skin, and distal nose were significantly different from each other. Greater similarities are evident between the conjunctival and nasal communities, which may suggest that either mucosal immunity or the anatomical connection of the nasolacrimal system impacts the microbial community populations of the canine conjunctiva and nasal microbiota. Further studies aimed at evaluating disease states and alterations of the canine conjunctival microbiota are warranted.

## Data Availability Statement

All 16S rRNA sequencing data have been deposited in the NCBI Sequence Read Archive under BioProject ID: PRJNA622265.

## Ethics Statement

This animal study was reviewed and approved by University of Missouri Institutional Animal Care and Use Committee. Written informed consent was obtained from the owners for the participation of their animals in this study.

## Author Contributions

KB participated in the conception and design of the study, sample collection, DNA extraction, data interpretation, and drafted manuscript. EG participated in the conception and design of the study, and manuscript revision. CR participated in the conception and design of the study, data interpretation, and manuscript revision. SB assisted with bioinformatics analysis and data management. AE participated in the conception and design of the study, DNA extraction, interpreted the sequence data, and helped to draft the manuscript. All authors read and approved the final manuscript.

## Conflict of Interest

KB resident in the MU College of Veterinary Medicine (CVM) Department of Veterinary Medicine and Surgery, is a veterinarian currently completing American Board of Veterinary Ophthalmology (ABVO) specialty training in veterinary comparative ophthalmology. EG Professor in the MU CVM Department of Veterinary Medicine and Surgery, is a veterinarian with specialty training in comparative ophthalmology and chief ABVO resident advisor to KB. CR Professor in the MU CVM Department of Veterinary Medicine and Surgery, is a veterinarian with specialty training in small animal internal medicine, and Director of the MU Comparative Internal Medicine Laboratory. AE Assistant Professor in the MU CVM Department of Veterinary Pathobiology, is a veterinarian with specialty training in laboratory animal and comparative medicine, and Director of the University of Missouri Metagenomics Center. SB Postdoctoral Researcher at the Luxembourg Centre for Systems Biomedicine, is a researcher with expertise in multi-omics integration, comparative medicine., and systems ecology. The authors declare that the research was conducted in the absence of any commercial or financial relationships that could be construed as a potential conflict of interest.

## Supplementary Material

Supplementary Figure 1 Chao1 **(A)** and Shannon **(B)** indices as estimates of true richness and α-diversity, respectively, generated using data subsampled to a uniform coverage of 1,461 sequences/sample. Bars indicate *p* < 0.05, Kruskal-Wallis ANOVA on ranks.

Supplementary Figure 2 Principal coordinate analysis showing the β-diversity within and between sample sites, as determined using Bray-Curtis **(A)** or Jaccard **(B)** similarities, and generated using data subsampled to a uniform coverage of 1,461 sequences/sample. Ovals represent 95% confidence intervals. Results of PERMANOVA are given on each plot; legend at right.

Supplementary Table 1 Patient demographics including age in years, gender, breed, weight (kg), height at the withers (cm), body condition (1 of 9/9 scale), and coat type. X—indicates a cross-bred dog with the breed listed. FS, Female, spayed; MC, Male castrated; MI, Male intact.

Supplementary Table 2 Aerobic culture results from conjunctival swabs of all subjects. Positive growth was detected in 11/25 samples.

Supplementary Table 3 List of all ASVs, and resolved taxonomies, identified in greater than half of samples from at least one site (gray cells). For each ASV, the prevalence within that site, and proportion of total DNA from that site represented by that ASV are provided. ASVs listed in gray cells were detected in greater than half of samples from all three sites.
