# Supplementary figures and images for: Evaluation of Healthy Canine Conjunctival, Periocular Haired Skin, and Nasal Microbiota Compared to Conjunctival Culture
Source: Front Vet Sci. 2020 Aug 27;7:558. doi: 10.3389/fvets.2020.00558 (PMC7481369; doi:10.3389/fvets.2020.00558)

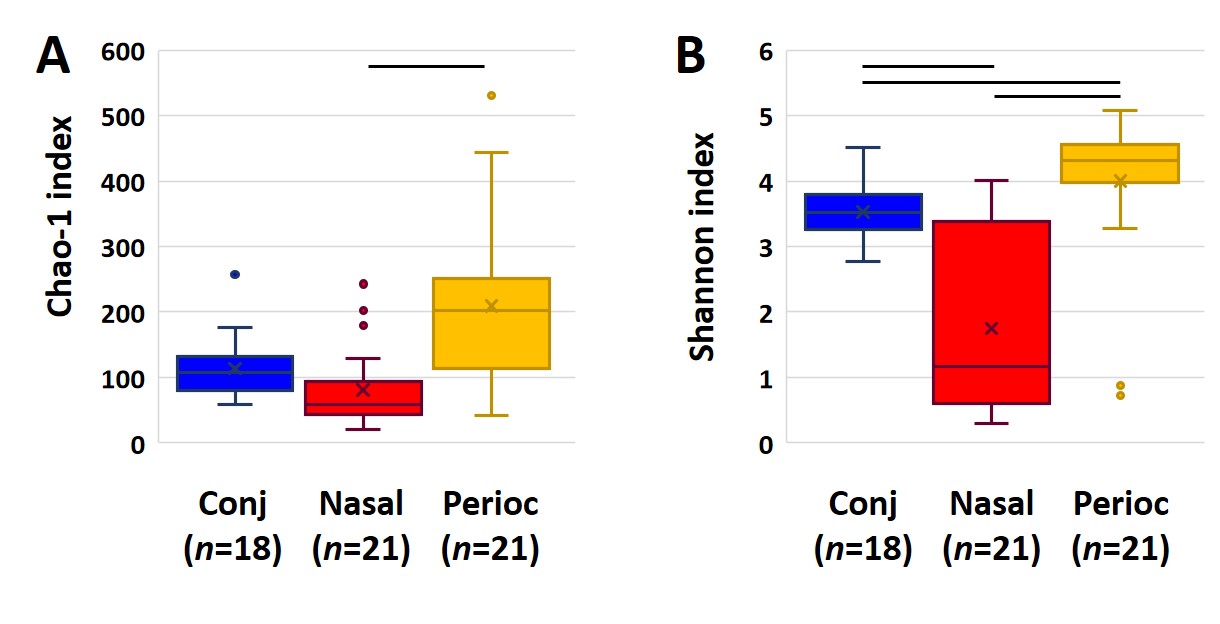

Supplement: Supplementary Figure 1 — Chao1 (A) and Shannon (B) indices as estimates of true richness and α-diversity, respectively, generated using data subsampled to a uniform coverage of 1,461 sequences/sample. Bars indicate p < 0.05, Kruskal-Wallis ANOVA on ranks. [file Image_1.JPEG]

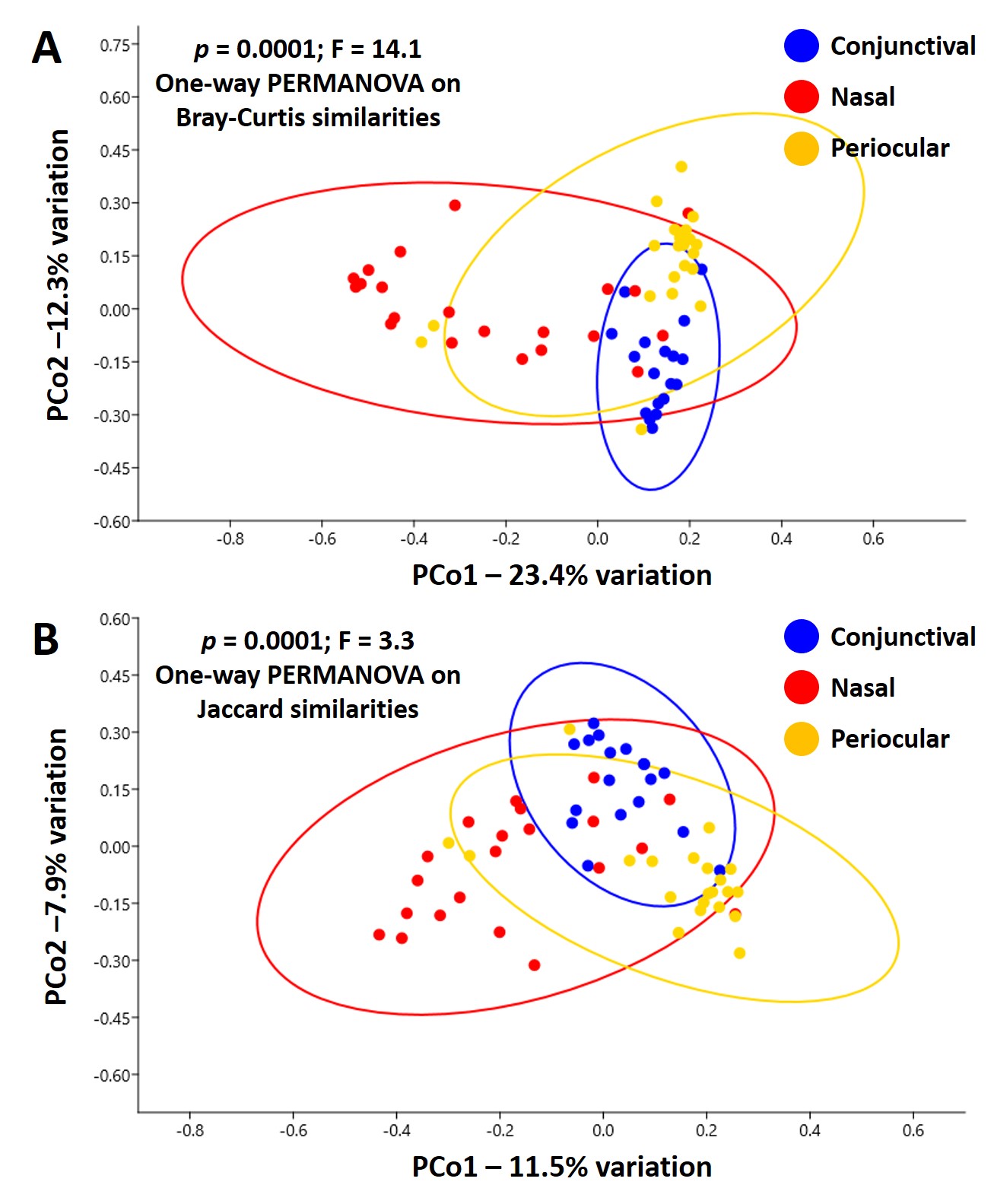

Supplement: Supplementary Figure 2 — Principal coordinate analysis showing the β-diversity within and between sample sites, as determined using Bray-Curtis (A) or Jaccard (B) similarities, and generated using data subsampled to a uniform coverage of 1,461 sequences/sample. Ovals represent 95% confidence intervals. Results of PERMANOVA are given on each plot; legend at right. [file Image_2.JPEG]
