# Supplementary material for: Evaluation of Healthy Canine Conjunctival, Periocular Haired Skin, and Nasal Microbiota Compared to Conjunctival Culture
Source: Front Vet Sci. 2020 Aug 27;7:558. doi: 10.3389/fvets.2020.00558 (PMC7481369; doi:10.3389/fvets.2020.00558)
Supplement: Supplementary Table 1 — Patient demographics including age in years, gender, breed, weight (kg), height at the withers (cm), body condition (1 of 9/9 scale), and coat type. X—indicates a cross-bred dog with the breed listed. FS, Female, spayed; MC, Male castrated; MI, Male intact. [file Table_2.DOCX]

| **Supplementary Table 1.** | | | | | | |
| --- | --- | --- | --- | --- | --- | --- |
| **Dog #** | **Age (yr)** | **Sex** | **Breed** | **Weight (kg)** | **Height (cm)** | **BCS (_/9)** |
| **1** | 2 | MC | Cattle dog X | 23 | 60 | 4 |
| **2** | 2 | FS | Border Collie X | 20 | 53 | 6 |
| **3** | 2 | FS | Golden retriever X | 25 | 59 | 6 |
| **4** | 5 | MC | Mixed breed | 25 | 57 | 5 |
| **5** | 5 | FS | Labrador X | 18 | 57 | 4 |
| **6** | 2 | FS | Mixed breed | 24 | 62 | 5 |
| **7** | 5 | FS | Catahoula leopard dog | 23 | 50 | 5 |
| **8** | 5 | MC | Mixed breed | 24 | 54 | 5 |
| **9** | 2 | FS | Cattle dog | 19 | 55 | 4 |
| **10** | 2 | FS | Mixed breed | 22 | 51 | 5 |
| **11** | 5 | MC | Mixed breed | 18 | 54 | 4 |
| **12** | 8 | FS | Mixed breed | 22 | 48 | 5 |
| **13** | 2 | MC | Mixed breed | 21 | 54 | 5 |
| **14** | 5 | MC | German Shepherd X | 24 | 51 | 6 |
| **15** | 5 | FS | Mixed breed | 19 | 54 | 5 |
| **16** | 6 | MC | Australian Shepherd | 17 | 48 | 5 |
| **17** | 5 | FS | Mixed breed | 19 | 54 | 5 |
| **18** | 1 | MI | Wirehair Vizsla | 24 | 65 | 3 |
| **19** | 1 | MI | Wirehair Vizsla | 25 | 66 | 4 |
| **20** | 7 | FS | Portuguese water dog | 22 | 55 | 5 |
| **21** | 3 | MC | Labrador | 25 | 54 | 5 |
| **22** | 4 | MC | Australian Shepherd | 24 | 58 | 6 |
| **23** | 2 | MC | Australian Shepherd | 20 | 56 | 4 |
| **24** | 5 | MC | Border Collie X | 19 | 56 | 5 |
| **25** | 1 | FS | Mixed breed | 17 | 52 | 4 |

**Supplementary Table 1.** Patient demographics including age in years, gender, breed, weight (kg), height at the withers (cm), body condition (1 of 9/9 scale), and coat type. * X – indicates a cross-bred dog with the breed listed. FS: Female, spayed; MC: Male, castrated; MI: Male, intact

| **Supplementary Table 2.** | | |
| --- | --- | --- |
| **Dog #** | **Culture Growth (Y/N)** | **Organism(s)** |
| **1** | Y, enrichment broth | *Paenibacillus terrigena* |
| **2** | Y, enrichment broth | *Staphylococcus epidermidis* |
| **3** | N |  |
| **4** | N |  |
| **5** | Y, enrichment broth | *Staphylococcus hominis* |
| **6** | N |  |
| **7** |  |  |
| **8** | Y, enrichment broth | *Bacillus pseudomycoides* |
| **9** | Y, enrichment broth | *Bacillus cereus; Staphylococcus pseudintermedius* |
| **10** | N |  |
| **11** | N |  |
| **12** | N |  |
| **13** | N |  |
| **14** | Y, enrichment broth | *Bacillus oceanisediminis* |
| **15** | N |  |
| **16** | N |  |
| **17** | Y, enrichment broth | *Staphylococcus epidermidis* |
| **18** | Y, enrichment broth | *Bacillus megaterium; Staphylococcus pseudintermedius* |
| **19** | Y, enrichment broth | *Bacillus cereus* |
| **20** | N |  |
| **21** | Y, enrichment broth | *Enterococcus faecium* |
| **22** | N |  |
| **23** | N |  |
| **24** | Y, light on direct culture | *Staphylococcus warneri* |
| **25** | N |  |

**Supplementary table 2.** Aerobic culture results from conjunctival swabs of all subjects. Positive growth was detected in 11/25 samples.
